# Polycomb-independent activity of EZH2 in castration resistant prostate cancer

**DOI:** 10.1186/1756-8935-6-S1-O14

**Published:** 2013-03-18

**Authors:** Kexin Xu, Zhenhua Jeremy Wu, Anna C Groner, Housheng Hansen He, Changmeng Cai, Edward C Stack, Massimo Loda, Tao Liu, Colm Morrissey, Robert L Vessella, Philip W Kantoff, Steven P Balk, X Shirley Liu, Myles Brown

**Affiliations:** 1 Center for Functional Cancer Epigenetics, Dana-Farber Cancer Institute, Boston, MA 02215, USA; 2 Department of Medical Oncology, Dana-Farber Cancer Institute and Harvard Medical School, Boston, MA 02115, USA; 3 Department of Biostatistics and Computational Biology, Dana-Farber Cancer Institute and Harvard School of Public Health, Boston, MA 02115, USA; 4 Hematology-Oncology Division, Department of Medicine, Beth Israel Deaconess Medical Center and Harvard Medical School, Boston, MA 02215, USA; 5 Center for Molecular Oncologic Pathology, Dana-Farber Cancer Institute, Boston, MA 02115, USA; 6 Department of Pathology, Brigham and Women’s Hospital, Harvard Medical School, Boston, MA 02115, USA; 7 Division of Cancer Studies, King’s College London, London SE18UB, UK; 8 Department of Urology, University of Washington Medical Center, Seattle, WA 98195, USA; 9 Puget Sound VA Health Care System, Seattle, WA 98108, USA

## 

Epigenetic regulators represent a new class of therapeutic targets for cancer [1]. Substantial studies suggest that the enhancer of zeste homolog 2 (EZH2) is one of such promising targets [2-4]. The current model of EZH2 oncogenic activity primarily focuses on its function as a subunit of Polycomb repressive complex 2 (PRC2), which silences gene expression via EZH2 histone methyltransferase activity [5,6].

Using a genome-wide approach we found that the oncogenic function of EZH2 in castration resistant prostate cancer (CRPC) is independent of its role as a transcriptional repressor. Instead, it involves the ability of EZH2 to act as a co-activator for critical transcription factors including the androgen receptor (AR). This functional switch is dependent on phosphorylation of EZH2, and requires an intact methyltransferase domain. Given that the loss-of-function mutations of EZH2 were observed in myelodysplastic syndrome and acute leukemia [7,8], our discovery of the non-PRC2 function of EZH2 in CRPC raises the potential to develop inhibitors that specifically target the EZH2 activation function while sparing its PRC2 repressive function to avoid the potential hematologic side effects. In addition, our finding that EZH2 cooperates with AR-associated complexes and requires phosphorylation to support CRPC growth suggests novel combination therapies for the treatment of metastatic, hormone-refractory prostate cancer.
